# Endothelial Protein C Receptor and Its Impact on Rheumatic Disease

**DOI:** 10.3390/jcm13072030

**Published:** 2024-03-31

**Authors:** Zachary Daniel O’Hehir, Tom Lynch, Sean O’Neill, Lyn March, Meilang Xue

**Affiliations:** 1 Sutton Arthritis Research Laboratory, Sydney Musculoskeletal Health, Kolling Institute, Faculty of Medicine and Health, The University of Sydney at Royal North Shore Hospital, Sydney, NSW 2065, Australia; zach.o@bigpond.com; 2 The Australian Arthritis and Autoimmune Biobank Collaborative (A3BC), Institute of Bone and Joint Research, Kolling Institute, Faculty of Medicine and Health, University of Sydney at Royal North Shore Hospital, St Leonards, NSW 2065, Australia; tom.lynch@sydney.edu.au (T.L.); lyn.march@sydney.edu.au (L.M.); 3 Department of Rheumatology, Royal North Shore Hospital, Syndey, NSW 2065, Australia; sean.oneill@sydney.edu.au

**Keywords:** endothelial protein C receptor, activated protein C, rheumatoid arthritis, systemic lupus erythematosus

## Abstract

Endothelial Protein C Receptor (EPCR) is a key regulator of the activated protein C anti-coagulation pathway due to its role in the binding and activation of this protein. EPCR also binds to other ligands such as Factor VII and X, γδ T-cells, plasmodium falciparum erythrocyte membrane protein 1, and Secretory group V Phospholipases A2, facilitating ligand-specific functions. The functions of EPCR can also be regulated by soluble (s)EPCR that competes for the binding sites of membrane-bound (m)EPCR. sEPCR is created when mEPCR is shed from the cell surface. The propensity of shedding alters depending on the genetic haplotype of the *EPCR* gene that an individual may possess. EPCR plays an active role in normal homeostasis, anti-coagulation pathways, inflammation, and cell stemness. Due to these properties, EPCR is considered a potential effector/mediator of inflammatory diseases. Rheumatic diseases such as rheumatoid arthritis and systemic lupus erythematosus are autoimmune/inflammatory conditions that are associated with elevated EPCR levels and disease activity, potentially driven by EPCR. This review highlights the functions of EPCR and its contribution to rheumatic diseases.

## 1. Introduction

The Endothelial Protein C Receptor (EPCR, CD201) is a transmembrane protein that is expressed by a variety of cell types in the human body, including myeloid cells, tissue-resident cells, and even platelets. It plays a vital role in mediating the anti-clotting and anti-inflammatory functions of a natural anticoagulant, activated protein C (APC) [1]. EPCR can also interact with various other ligands, eliciting ligand-dependent functions. Additionally, EPCR is homologous to MHCI/CD1 family proteins [2], with the potential to regulate both innate and adaptive immunity. Moreover, EPCR has been identified as a promising stem cell marker for multiple cell types in both humans and mice. It is worth noting that EPCR’s functions can be affected by its soluble form (sEPCR), which competes for the binding sites of membrane-bound (m)EPCR. sEPCR is created when mEPCR is shed from the cell surface, and its circulating level depends mainly on the genetic haplotype of the *EPCR* gene that an individual may possess. Higher levels of EPCR have been found in many inflammatory/autoimmune diseases and may contribute to disease pathogenesis. This review summarises the functions of EPCR and its contribution to rheumatic diseases.

## 2. Endothelial Cell Protein C Receptor

**Figure 1:**
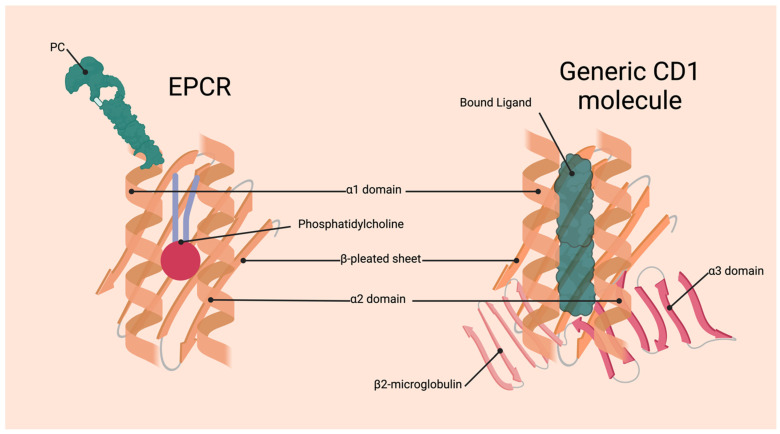
The structures of EPCR vs. CD1. The EPCR molecule forms a deep hydrophobic groove between two anti-parallel α-helices, the α1 and α2 domains, sitting atop a β-pleated sheet that houses the phospholipid phosphatidylcholine. This lipid is bound to EPCR and assists in its binding to PC by maintaining the structure of EPCR. The ligands of EPCR do not bind inside the hydrophobic groove [7]. The group of CD1 proteins possesses a similar structure to EPCR; however, they possess an α3 domain that associates with β2-microglobulin. The deep grooves formed from these structures are used for ligand binding in the CD1 molecules and can vary between each of the CD1 molecules depending on the structure of the groove [8].

The human *EPCR* gene is located at position 20q11.2 with a length of 8 kb and comprises four exons separated by three introns [3,4]. The 5′ untranslated region and a single peptide is coded by exon 1, the extracellular domain is coded by exon 2 and 3, and exon 4 is responsible for the 3′ untranslated region as well as the transmembrane domain and the cytoplasmic tail [4]. The encoded human EPCR protein is an N-glycosylated type 1 membrane protein. It has a similar 3D representation to the MHCI/CD1 proteins; however, a characterising deep groove that is usually required for antigen binding instead contains a lipid in EPCR [1,5]. In most cases, this lipid will be phosphatidylcholine; however, recent studies have found a variety of lipids that can be bound to EPCR with varying frequency [6]. The bound lipid contributes to the binding of EPCR to Protein C (PC), likely by maintaining the structure of EPCR rather than through direct contact [1,7] (Figure 1). 

Human EPCR has also been found to bear a great homology of 62% to murine protein CCD41 as they come from the same gene and are altered through post-transcriptional or post-translational processes [9,10]. The proteins are distinguished by their unique cell position. CCD41 is predominantly located in the centrosome close to the nucleus and in the perinuclear vesicles during cell cycle progression and, as a result, can only indirectly interact with APC. EPCR has both surface cell receptor and soluble forms that can directly bind to APC, unlike CCD41 [11,12].

### 2.1. EPCR Expression and Localisation

EPCR expression was originally detected in the endothelium of large blood vessels [13,14] and the microvascular endothelium of the lungs, heart, and skin to a lesser extent [15]. Subsequently, other cell types found to express EPCR included neutrophils, natural killer cells, monocytes, keratinocytes, smooth muscle cells, cardiomyocytes, eosinophils neurons, placental trophoblasts, and dendritic cells [16,17,18,19], mucosal tissues such as the lung and gut, and joint synovial fibroblasts [20]. Additionally, hematopoietic, epithelial, neuronal, and multipotent progenitor cells; breast cancer stem cells; and skin epidermal stem cells express EPCR, and EPCR acts as a potential stem cell marker for these cells [21,22,23,24,25,26,27,28]. 

EPCR on the cell surface, also known as mEPCR, is predominantly localised in membrane microdomains such as caveolin-1. EPCR can also be detected in the endosomes and colocalised with lysosome protein marker 1 in smaller quantities [14]. Through endocytosis and recycling of ligand-bound surface EPCR, the cell can internalise certain ligands through a dynamin and caveolar-dependent pathway such that they can be translocated to extravascular tissues, thereby impacting ligand bioavailability [14,29]. This process is regulated in a temporospatial manner by specific Ras-like small GTPases [30]. Intracellular EPCR has been found in endothelial cells, mostly around the perinuclear region, with some dispersed throughout the cytoplasm [14]. 

### 2.2. EPCR Function 

EPCR is crucial in maintaining normal homeostasis and regulating coagulation and inflammation. Most of the experimental evidence on the physiological and pathological importance of EPCR is derived from studies on gene-modified mice. 

#### 2.2.1. Regulation of Normal Homeostasis

Deletion of the *EPCR* gene causes early embryonic lethality in mice [31]. Further investigation revealed critical EPCR expression on trophoblast giant cells [31]. Mice with EPCR expression on placenta giant trophoblasts being carried to term develop normally and are healthy [32]. Mice with a variant of EPCR that cannot bind to PC/APC develop splenomegaly due to bone marrow (BM) failure [33], a phenotype that was not observed in the aforementioned EPCR-deficient mice [32]. BM transplant experiments suggest that EPCR modulates haematopoiesis [33].

#### 2.2.2. Regulation of Coagulation 

EPCR is known to promote the activation of PC to APC on the cell surface [34] and regulate the anti-coagulation pathways. In mice, disrupting the *EPCR* gene can lead to fibrin deposition and placental thrombosis [31]. As EPCR-deficient mice age, they generate more thrombin and activate less PC in response to procoagulant stimuli, resulting in increased spontaneous thrombin formation [32]. In a mouse model of haemophilia, this EPCR deficiency is responsible for the reduced intra-articular bleeding initially and the reduced severity of related arthropathy in the longer term [35]. Furthermore, mice with an EPCR variant that cannot bind to PC/APC have impaired activation of PC and increased generation of thrombin in response to thrombotic and inflammatory challenges compared to wild-type mice [33].

#### 2.2.3. Regulation of Inflammation

EPCR plays a crucial role in controlling inflammation [1]. Overexpression of EPCR protects mice from endotoxin-induced injury [36], and EPCR-deficient mice displayed a higher mortality rate, more thrombin generation, platelet consumption, and immune cell sequestration in the lung tissue in response to lipopolysaccharide (LPS) [37]. These mice are more susceptible to dextran sulphate sodium -induced colitis, manifested by inflammation and mucosal barrier disruption [38]. These findings are consistent with previous studies on EPCR low-expression mice [39] and baboons treated with EPCR-blocking antibodies [40]. EPCR can inhibit Th17 cells, and T cell-specific EPCR deficiency exacerbates experimental autoimmune encephalomyelitis in mice [41]. Paradoxically, EPCR on circulating T cells positively correlates with disease activity in psoriasis [42]. Similarly, elevated levels of EPCR can predict poor outcomes of severe lung infection and inflammation [43], colorectal and lung cancers [44,45], and lupus nephritis patients [46]. Conversely, EPCR deficiency ameliorates murine inflammatory arthritis, protects against bacterial-induced lung injury [47], inhibits joint bleeding-induced inflammation [35], and deters the development of lupus and antiphospholipid syndrome [48] in mice. The underlying mechanisms of EPCR’s conflicting functions in different diseases are not clear but may be associated with its ligands present on specific cells/organs. 

#### 2.2.4. Regulation of Stemness 

EPCR has been identified as a promising stem cell marker for several cell types. It is a dependable marker of long-term haematopoietic stem cells (HSCs) [49] and can be used to specifically identify HSCs in murine BM [50]. EPCR also marks human foetal liver HSCs [51]; human epidermal stem cells [28]; human cord blood HSCs [25]; and progenitor cells of the endothelium [52], neuron [53] and epithelium [54,55]. EPCR is also expressed by highly aggressive basal-like breast cancer subtypes. In aggressive triple-negative breast cancer cells, EPCR expression is a characteristic of cancer stem cell-like populations with tumour-initiating properties in vivo [56]. Interestingly, the functions of EPCR in coagulation and inflammation are mainly controlled by EPCR on non-hematopoietic cells [37,57]. Selective deletion of non-hematopoietic EPCR almost completely abolishes PC activation [37]. 

### 2.3. EPCR Function Mediators

#### 2.3.1. Soluble EPCR 

**Figure 2:**
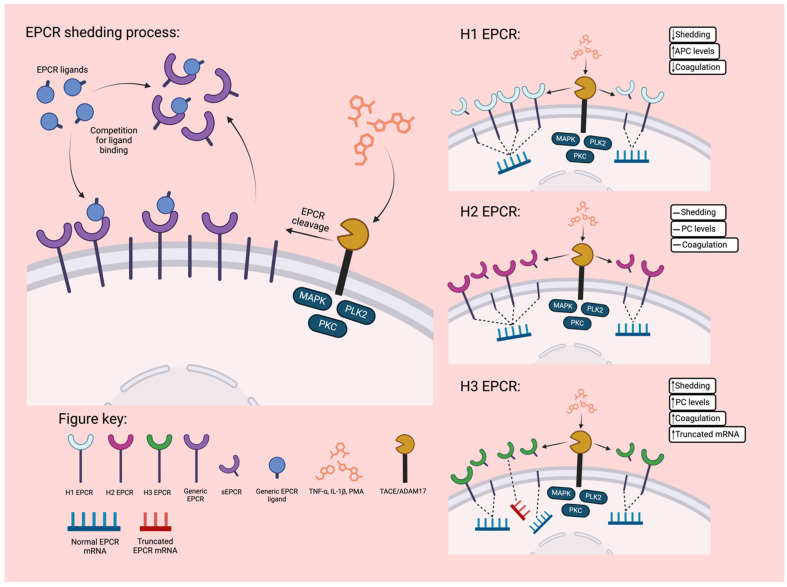
Regulation of EPCR shedding. mEPCR is cleaved from the cell surface through a process mediated by TACE to create sEPCR. TACE cleavage is regulated by Polo-like kinase 2 (PLK2), mitogen-activated protein kinases (MAPKs), and protein kinase C (PKC) [91] and can be induced by pre-treatment of cells with PMA, TNF-α, or IL-1β [60,61,62,63]. Once cleaved, the mEPCR and sEPCR will compete for ligand binding, making fewer mEPCR-ligand functions realised [64]. H1 favours mEPCR compared to the common allele of H2 due to decreased TACE cleavage. In contrast, H3 favours sEPCR via increased TACE cleavage and increased production of truncated mRNA that creates a functionally similar isoform of sEPCR [59,67,77,78].

Cell surface EPCR can be shed through a process mediated by TNF-α converting enzyme/ADAM17 (TACE) [58] or via alternative mRNA splicing [59] to generate sEPCR. Additionally, pre-treatment of samples with phorbol-12-myristate 13-acetate (PMA), TNF-α, IL-1β, or LPS induces EPCR shedding (Figure 2) [60,61,62,63]. sEPCR does not have the transmembrane and cytoplasmic tail domain of mEPCR [64], but it can bind both PC and APC with an affinity similar to mEPCR. Binding PC to sEPCR rather than mEPCR blocks surface interactions with negatively charged phospholipids that are required for the efficient inactivation of factor (F)V and FVIIIa. This binding also inhibits the activation of PC to APC, thus interfering with the anti-inflammatory and anti-coagulative effects of EPCR [65,66,67]. High plasma sEPCR can result in a low mEPCR level and reduced APC activity, leading to a pro-thrombotic state in the body [68]. Plasma sEPCR, therefore, has the potential as a marker for hypercoagulable states [64,69]. 

#### 2.3.2. Anti-EPCR Autoantibodies

High levels of anti-EPCR autoantibodies have been observed in patients with antiphospholipid syndrome (APS) [70], a condition that is linked with thrombosis and foetal death. These autoantibodies sometimes obstruct the binding of PC to EPCR, thus hampering the generation of APC [70]. Recently, anti-EPCR autoantibodies have also been detected in patients with Takayasu arteritis—a type of large vessel vasculitis. Interestingly, patients with these autoantibodies were more prone to strokes [71]. Additionally, more than 60% of patients with primary ulcerative colitis (UC) tested positive for anti-EPCR autoantibodies [71,72], which suggests that these antibodies may prove useful in diagnosing UC. In the general population, the presence of anti-EPCR autoantibodies is a moderate risk factor for deep vein thrombosis [73]. Among young women, anti-EPCR autoantibodies have been associated with acute myocardial infarction [74] and are independent risk factors for foetal death [70].

#### 2.3.3. EPCR Genetic Variants 

The *EPCR* gene has several functional genetic mutations that lead to reduced expression of EPCR or receptor dysfunction. Such mutations may be the result of a rare 23-bp insertion in exon 3 [75] or a variety of single-nucleotide polymorphisms (SNPs) in coding or non-coding regions of the *EPCR* gene. There are sixteen SNPs present in the *EPCR* gene, which can be divided into four haplotypes (H1–4) [67,76,77].

H1 contains the combination of 10 specific alleles that have been altered from the common alleles of the H2 haplotype. These are the 1451T (rs2069943), 1541A (rs2069944), 1880C (rs2069945), 2532C (rs2069948), 2897A (rs945960), 3424C (rs871480), 3997C (rs2069952), 4678C (rs9574), 5632G (rs1415773), and 5663A (rs1415774) alleles. This haplotype causes an increase in mEPCR that subsequently increases the levels of APC and provides anti-coagulative effects (Figure 2) [78,79]. The specific SNP responsible for this increased level of mEPCR is currently unknown.

The H3 haplotype is tagged by the g.4600A>G minor allele (Ser219Gly; rs867186), with the rs867186-GG genotype, in particular, being responsible for the increased production of plasma sEPCR [67,77,79,80,81,82,83,84], causing an increase in risk for pro-coagulative disease states such as venous and arterial thrombosis, deep vein thrombosis, and miscarriage [65,67,85,86,87]. This mutation predicts an amino acid change in Ser219Gly in the receptor’s transmembrane region, facilitating the removal of EPCR from the cytomembrane via metalloproteinases (MMPs). Furthermore, it is associated with a truncated form of EPCR mRNA that lacks the transmembrane and intracellular domains and is likely responsible for additional cleavage of mEPCR to sEPCR (Figure 2) [59]. SNP rs867186 explains ~85% of the phenotypic variance [82] and is responsible for elevated levels of plasma sEPCR during inflammatory disorders. The frequency of Ser219Gly polymorphism in the general population is about 12% [83]. EPCR H3 is also associated with an increase in the levels of plasma PC that is speculated to be caused by a decrease in mEPCR levels and EPCR-dependent activation of PC due to excess shedding [79,80,88,89]. Similarly, higher levels of plasma FVII are associated with the H3 haplotype [90].

Finally, the H2 haplotype is the set of common alleles for the gene [67,77], while H4 rarely occurs and is associated with a slight increase in the risk of venous thromboembolism [77].

## 3. EPCR Ligands and Ligand Associated Functions

Many functions of EPCR depend on its interaction with a variety of ligands. A previous report showed that EPCR was constitutively translocated into the nucleus [92], indicating that EPCR may internalise its ligands and translocate them into the nucleus, which could directly influence gene expression [14]. Below are some of the ligands that EPCR binds to and their resulting outcomes upon binding.

### 3.1. Activated Protein C

**Figure 3:**
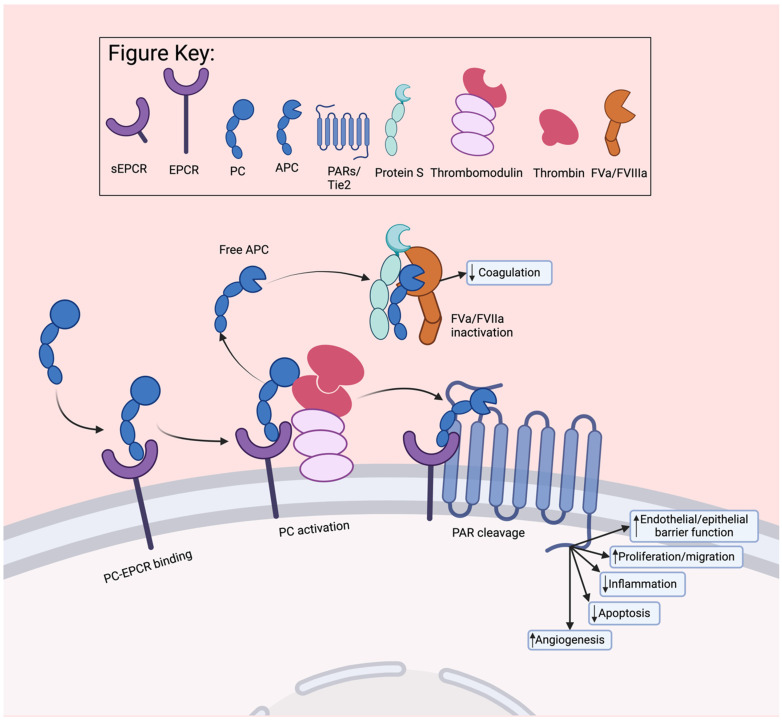
EPCR-PC pathways. When PC is bound to EPCR the activation of PC by thrombin–thrombomodulin complex is increased by approximately 20-fold [95,96]. Once activated, APC will either dissociate from EPCR to directly inactivate coagulation factors FVa or FVIIa with the assistance of PS [94] or APC will remain bound to EPCR to illicit cytoprotective functions via cleavage and activation of PARs [93].

The PC/APC pathway provides several cytoprotective properties, including anti-clotting, anti-inflammatory, anti-apoptotic, and barrier-protective functions [93]. The binding of thrombomodulin to thrombin activates PC to APC, which in turn allows for the inactivation of FV and FVIII via a complex made of APC, Protein S (PS), phospholipids, and calcium [94] (Figure 3). The binding of EPCR to PC enhances the activation of PC by 20-fold, thus providing an anti-coagulation effect [34,95,96]. Blocking EPCR binding to PC accelerates thrombus development in a murine model of thrombosis, confirming that EPCR plays a crucial role in regulating coagulation [64,97]. EPCR-deficient mice are significantly protected from acquired haemophilia, suggesting that the EPCR-mediated APC anticoagulant pathway plays a critical role in haemophilia [98]. 

After being activated, APC can either dissociate from EPCR and perform anticoagulant functions or remain bound to EPCR and exhibit cell-signalling cytoprotective activities (Figure 3) [1]. When bound to EPCR, APC cleaves PAR1 at Arg-46, which triggers β-arrestin-2 biased PAR1 signalling [99,100,101,102] and exerts its anti-apoptotic, anti-inflammatory, and barrier stabilising functions [103,104,105]. Additionally, when bound to EPCR, APC elicits biased cytoprotective signalling through the cleavage of PAR3 at the Arg41 noncanonical site [49,106]. Moreover, the interaction of APC with EPCR, PAR3, and neutrophil-expressed Mac-1 generates the signalling required to inhibit PMA-induced neutrophil extracellular trap (NET) formation [107]. The APC-EPCR signalling pathways are the main mechanisms for APC’s beneficial effects in various autoimmune and inflammatory diseases, including type 1 diabetes [108], inflammatory arthritis [109], systemic lupus erythematosus (SLE), and lupus nephritis (LN) [110].

### 3.2. Factor VII

**Figure 4:**
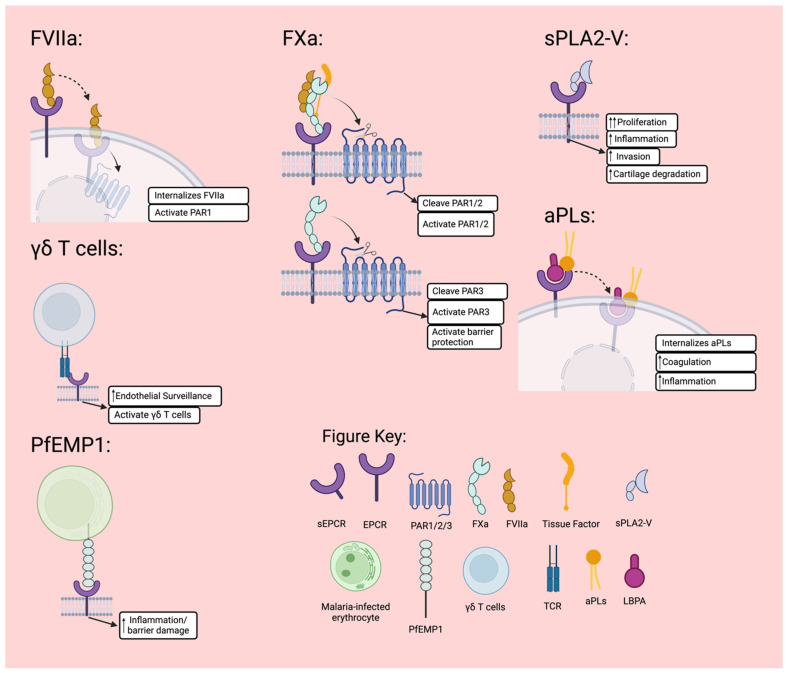
Ligand function summary. EPCR binds to FVII/FVIIa with similar affinity to APC and will compete with APC for ligand binding. When bound to EPCR, FVIIa will be internalised with EPCR and activate endogenous PAR1. FXa can bind to EPCR in a TF-FVIIa-FXa complex, or it can bind to EPCR by itself. The TF-FVIIa-FXa will incite PAR2 activation, which is necessary for TLR4 and interferon regulation host defences [117]. By itself, FXa bound to EPCR will cleave and activate PAR3 to prolong activation of the barrier-protective tunica intima endothelial receptor tyrosine kinase 2 [119]. γδ T cells bind to EPCR at a different site to APC and will cause a potential increase in endothelial surveillance for viral infections or malignancies [122,123]. PfEMP1 on malaria-infected erythrocytes competes for the APC binding site on EPCR [124]. It is associated with the severity of malaria [125]. sPLA2-V competes with APC for EPCR binding and mediates the aggressive behaviour of synovial fibroblasts from rheumatoid arthritis (RA) [127,129]. aPLs undergo internalisation when bound to EPCR. The lipid bound in the groove of EPCR is phosphatidylcholine in most cases. However, it can be exchanged with LBPA, resulting in an EPCR-LBPA complex that facilitates the endosomal trafficking of aPLs [48,137].

EPCR may exhibit APC/PC-independent anticoagulant activities via FVII interaction. Human recombinant FVIIa in pharmacological concentrations inhibits EPCR-mediated APC production on endothelial cells, suggesting competition between FVIIa and PC/APC for EPCR binding [98,111]. Further studies have confirmed that EPCR is a receptor for FVII, FVIIa, PC, and APC with similar affinity [14,64]. When bound to EPCR, FVIIa is internalised, which could indicate that EPCR assists FVIIa translocation and signalling pathways (Figure 4) [64]. Like APC, FVIIa bound to EPCR does not modulate thrombin-induced endothelial barrier permeability [112]; rather, it elicits a PAR1 and β-arrestin-1 dependent anti-inflammatory signalling pathway [113]. This binding attenuates LPS-induced vascular leakage in the mouse lung and kidney [114]. Furthermore, PAR1-mediated p44/42 mitogen-activated protein kinase (MAPK) activation is induced upon EPCR binding and activation of endogenous PAR1 [113]. Additionally, the binding of EPCR and FVIIa downregulates the production of APC and contributes to a haemostatic effect in haemophilia [98]. 

### 3.3. Factor X

FX is a zymogen that plays a significant role in the coagulation cascade. Once activated to FXa, it competes with APC for the binding site of EPCR in a calcium-dependent manner [115]. When bound to EPCR, FXa cleaves and activates PARs, which are crucial for the activation of coagulation pathways. Activation of PAR1 through FXa depended on the EPCR expressed in Chinese hamster ovary cells [116]. EPCR promotes efficient cleavage of PAR1 and PAR2 by the Tissue Factor (TF)-FVIIa-FXa complex (Figure 4); however, it does not change the efficacy of PAR2 cleavage by TF-FVIIa [49]. Furthermore, EPCR activates PAR2 via the ternary TF-FVIIa-FXa [117], acting as a necessary coreceptor in cell signalling. This is further supported by EPCR-dependent TF-FVIIa-FXa-mediated activation of the p44/42 MAPK signalling cascade [118]. Moreover, EPCR greatly improves the efficacy of noncanonical PAR3 cleavage by FXa (Figure 4). This FXa-EPCR-induced PAR3 cleavage results in prolonged activation of the barrier-protective tunica intima endothelial receptor tyrosine kinase 2 [119]. 

### 3.4. γδ T Cells

γδ T cells are important for immunity at barrier surfaces [120]. EPCR has been identified as a ligand for the subpopulation of T lymphocytes that bear the γδ T-cell antigen receptor [93,121]. A subpopulation of γδ T cells called Vδ2¯− γδ T cells can recognise cytomegalovirus-infected cells through binding to EPCR. The β sheet of EPCR is the binding site for this subpopulation of γδ T cells, and the binding is dependent on conformational integrity instead of lipid binding [122]. This binding of γδ T cells to EPCR may play a role in the surveillance of endothelium for viral infections or malignancies (Figure 4) [122,123].

### 3.5. Plasmodium Falciparum Erythrocyte Membrane Protein 1 (PfEMP1)

PfEMP1 is found on the surface of erythrocytes infected by *Plasmodium falciparum*. During malaria infection, PfEMP1 will compete with APC for the binding site of EPCR, resulting in the cytoprotective activation of PAR1 not being achieved (Figure 4) [124]. Furthermore, during malaria infection, EPCR is a major host receptor for the sequestration of *Plasmodium falciparum*-infected erythrocytes in the brain and other vital organs. The increased expression of EPCR-binding PfEMP1 is associated with progressively more severe disease [125]. 

### 3.6. Secretory Group V Phospholipases A2 (sPLA2V)

Phospholipases A2 are part of a superfamily of enzymes responsible for catalysing glycerophospholipids’ hydrolysis [126]. sPLA2V inhibited the generation and anti-apoptotic effects of APC by binding to EPCR on the endothelial cells (Figure 4) [127,128]. The binding of sPLA_2_V to EPCR also mediates the aggressive behaviour of RA synovial fibroblasts [129]. The binding occurs by exchanging phosphatidylcholine for lysophosphatidylcholine in the hydrophobic groove 

### 3.7. Proteinase-3 (PR3)/Macrophage-1 Antigen (Mac-1)

PR3, also named myeloblastin, is mainly recognised for its role in breaking down enzymes and structural proteins. It is stored within the azurophilic granules of polymorphonuclear neutrophils alongside cathepsin G and neutrophil elastase [130,131]. When neutrophils are activated, PR3 is released, which binds activated neutrophils to sEPCR [132,133]. Additionally, PR3 plays a role in the degradation of EPCR as it cleaves EPCR at multiple sites. Further studies have revealed that β2 integrin Mac-1 forms a heteromeric complex with PR3, suggesting that Mac-1 contributes to the binding between sEPCR and PR3 [134]. However, studies have shown that EPCR can bind to Mac-1 directly on monocytes and share the same site as APC binding [134,135,136]. 

### 3.8. Autoantibodies to Phospholipids

aPLs are a category of antibodies that target proteins that bind to phospholipids and are associated with various inflammatory/autoimmune diseases, including antiphospholipid syndrome (APS), SLE, and RA. These antibodies can activate coagulation pro-inflammatory pathways. A recent study discovered that EPCR is a cell surface target for aPLs [48]. In APS and SLE, the pathogenic effects of aPLs appear to be mediated by their interaction with EPCR. The lipid-bound in the groove of EPCR is phosphatidylcholine in most cases. However, it can be exchanged with lysophosphatidic acid (LBPA), resulting in an EPCR-LBPA complex that facilitates the endosomal trafficking of aPLs [48,137]. EPCR has been shown to interact with aPLs to activate trophoblast cells, monocytes, and dendritic cells. Monocytes and trophoblasts produce more pro-inflammatory cytokines (TNF and type 1 IFN) when aPLs bind to EPCR, which leads to the internalisation of aPLs (Figure 4) [48,137]. 

These observations indicate that EPCR may play a broader role in influencing various pathophysiological processes by interacting with different ligands in different milieus.

## 4. EPCR in Rheumatic Diseases

Rheumatic diseases refer to a group of autoimmune conditions that cause inflammation and pain in the joints, muscles, or fibrous tissues. EPCR plays a potential role in the development and ongoing activity of these debilitating conditions due to its role as a mediator of coagulation and inflammatory pathways. This review summarises current knowledge of EPCR functions in rheumatic diseases, focusing on RA and SLE. 

### 4.1. EPCR and RA

RA is one of the most prevalent chronic inflammatory diseases, affecting ~0.5–1% of the population, predominantly women. It primarily affects synovial joints but also impacts other organ systems [138], resulting in disability and premature death. The inflammation and tissue damage in RA are mediated by a coordinated response from the innate and adaptive immune system, including inflammatory cells and their autoantibody and cytokine production [139,140]. Currently, 269 SNPs have been identified as risk genes for RA development [141]. The gene with the closest correlation to RA disease progression is the human leukocyte antigen DRB1*04 epitope, which is shared by 80% of RA patients [142,143,144].

EPCR is significantly elevated in the synovium of patients with RA, while it aids APC in suppressing RA monocyte activation and migration [20]. This points to the possibility of EPCR having a protective role in mediating inflammatory states in RA. Additionally, it has been found that higher concentrations of EPCR may be associated with anti-inflammatory and protective effects due to its essential role in creating APC [99,145]. In fact, mice models that were treated with APC showed a decrease in arthritis severity by approximately 50% and a reduction in disease incidence by 40% [109].

Despite the cytoprotective properties of EPCR through the APC pathway, EPCR is observed to promote inflammatory responses by interacting with other ligands or through sEPCR. In RA synovial fibroblasts, EPCR promotes an inflammatory response through interacting with sPLA2V. This ligand competes with APC for the same ligand binding site [129], which interferes with regular APC-EPCR interactions and stimulates RA synovial proliferation and destruction [109]. This may also contribute to the production of sEPCR since the sera of arthritic mice have significantly higher levels of sEPCR than healthy controls [109,146]. Short-cut mRNA is increased in RA patients compared to osteoarthritis patients, indicating that variable EPCR mRNA splicing plays a key role in the overproduction of sEPCR in RA patients [146].

EPCR is known to interact with T cells and neutrophils in patients with RA, which can have both protective and destructive effects. In RA patients, lower levels of CD4+ EPCR+ T cells are observed compared to patients with osteoarthritis. This may lead to an increase in pathogenic Th17 cell differentiation in RA patients [146]. Contrarily, mice with severe EPCR deficiency displayed more than 40% reduced arthritis incidence and 50% decreased disease severity when compared to normal mice [147].

A potential mechanism for how EPCR may regulate RA is through the γδ T cell ligand. The concentration of γδ T cells is higher in human RA patients [148,149], and the percentage of Vδ2 T cells was negatively correlated to RA disease activity [121]. Another mechanism could involve NETs; the expression of NETs appears to increase when treated with EPCR-neutralising antibodies. Therefore, EPCR may regulate the secretion of NETs in RA patients through the APC-EPCR signalling pathway, thereby affecting the progression of disease in RA patients [150].

### 4.2. EPCR and SLE

SLE is a potentially fatal, chronic, multisystem autoimmune disorder that typically affects women between puberty and menopause. The disease is characterised by an abnormal response of the immune system involving B cells, T cells, dendritic cells, and macrophages [151]. The exact pathogenesis of the disease is still unknown; however, research suggests that environmental, genetic, and epigenetic factors may play a role in the development and progression of SLE. Currently, there are 60 genes identified as risk factors for SLE [151]. 

Several studies have observed increased sEPCR levels in SLE patients [152,153,154]. The severity of the disease and the use of corticosteroid treatment can impact the levels of sEPCR in SLE patients [152]. An individual’s EPCR haplotype may also play a role in the development of SLE, as the H3 haplotype is more commonly observed in SLE patients than in healthy controls. However, some patients with elevated sEPCR levels still have the H1, H2, and H4 haplotypes [153]. Moreover, complications of SLE, such as lupus nephritis (LN), can also affect the levels of EPCR. While SLE causes elevated sEPCR levels, patients with LN have significantly higher levels of sEPCR than patients with SLE who do not develop LN [155,156,157]. The increase in sEPCR levels in LN patients may be explained by the conversion of elevated mEPCR levels, which are found in the cortical peritubular capillaries of these patients, to sEPCR due to the shedding by the inflammatory cytokines IFN-γ and IL-1 [46,155]. 

APS is an autoimmune disease that often occurs in patients with SLE. It is characterised by repeated blood clots and is associated with the presence of aPLs. aPLs can be specifically recognised by the EPCR-LBPA complex on cell surfaces. This recognition can activate trophoblast cells, monocytes, and dendritic cells, leading to the development of APS autoimmunity [48]. In mice, blocking the EPCR-LBPA signalling pathway can prevent the development of APS, SLE-like syndrome, and kidney pathology associated with SLE [48]. Therefore, EPCR-LPBA signalling is a potential central mechanism for the development of SLE-like APS-related autoimmune diseases. 

### 4.3. EPCR and Other Rheumatic Diseases 

Scleroderma describes a group of rare autoimmune diseases that cause the hardening and tightening of skin. Arthritis and tendinopathy are common developments of scleroderma. In scleroderma, there is a downregulation of endothelial EPCR by Fli1 that could induce a hypercoagulable state, leading to tissue fibrosis and disruption of peripheral circulation [158]. 

Multiple Sclerosis (MS) is an autoimmune disease that affects the central nervous system and is associated with an increase in the subsequent diagnosis of RA [159]. Like RA, there are elevated levels of sEPCR in MS patients, which exceed that of SLE patients [160]. 

Haemophilic arthropathy (HA) is a debilitating joint condition. A murine study showed that mice with EPCR deficiency were afflicted with less severe HA due to reduced joint bleeding from lack of EPCR. The therapeutic, recombinant FVIIa was also more effective at preventing HA in the EPCR-deficient mice. EPCR deficiency is thought to have reduced the severity of HA by reducing the recurrence of spontaneous joint bleeding. It is also possible that the lack of APC generation results in enough thrombin generation to prevent further joint bleeding in HA [35].

## 5. Conclusions

EPCR is a transmembrane protein with various functions in several disease states, its most prominent contribution being its involvement in the PC/APC pathway. It is regulated by competition for its binding site between its membrane-bound and soluble form. The propensity for EPCR to come in either of these forms can vary depending on the haplotype of the *EPCR* gene, with H3 producing more sEPCR and H1 maintaining more mEPCR. In this review, we identified PC/APC, FVIIa, FXa, γδ T cells, PfEMP1, sPLA2V, Mac-1, PR3, and aPLs as ligands that can bind to EPCR, each of them providing unique cytoprotective or destructive effects. Many of these ligands have cytoprotective properties when bound to EPCR, such as through the cleavage of PARs; however, ligands such as sPLA2V and PfEMP1 have destructive properties when bound to EPCR. EPCR can regulate immune function by inhibiting Th17 cells and is useful as a marker for stem cells such as human epidermal stem cells or HSCs.

## 6. Clinical Implications and Future Perspectives

EPCR is a highly flexible protein that is expressed by different cell types and plays a vital role in coagulation, inflammation, and haemostasis. These key properties of EPCR make it a potential biomarker and target for treating various diseases. Among the rheumatic diseases, RA and SLE stand out as having significant associations with EPCR. In both SLE and APS, the binding of aPLs with the EPCR-LBPA complex induces pathogenic effects. Disrupting the formation of the EPCR-LBPA complex on the cell surface or preventing aPLs from binding to the EPCR-LBPA complex could be an effective approach for EPCR-targeted therapy for SLE and APS. The same mechanism may apply to RA, although there is no confirmation yet. However, studies have shown that mice with severe EPCR deficiency had a much lower disease incidence and milder arthritis [147], indicating that EPCR could also be a potential therapeutic target for RA. Therefore, it is essential to validate these findings in large clinical cohorts to assess EPCR function and its potential as a therapeutic target for rheumatoid diseases.

To create therapeutics that target EPCR for rheumatoid diseases, it is important to design them in a way that preserves EPCR’s ability to bind with other ligands, especially with PC/APC. This is necessary to maintain the anti-coagulation and anti-inflammatory properties of APC, as APC has been shown to have beneficial effects on inflammatory arthritis [109], SLE, and LN [110]. However, targeting EPCR may have both positive and negative effects, given its well-established functions in homeostasis, coagulation, inflammation, and stemness. 

It is still uncertain how exactly EPCR contributes to pathogenesis in rheumatic diseases, and the role of other ligands such as sPLA2V, PR3, Mac-1, γδ T cells, FVIIa, and FXa in these diseases has not been fully explored. Therefore, future research should aim to expand our knowledge of how these ligands interact with EPCR in order to develop targeted adjunctive therapies for rheumatic diseases.
